# Association between benign prostate enlargement-related storage and voiding symptoms and systolic blood pressure: a single-center cross-sectional study

**DOI:** 10.1590/1516-3180.2018.0543.R3.160919

**Published:** 2020-01-13

**Authors:** Eşref Oğuz Güven, Ismail Selvi, Eda Karaismailoğlu

**Affiliations:** I MD. Associate Professor, Department of Urology, Ankara Oncology Training and Research Hospital, Ankara, Turkey.; II MD. Urologist, Department of Urology, Karabük Üniversitesi Eğitim ve Araştırma Hastanesi, Karabük, Turkey.; III PhD. Research Assistant, Department of Biostatistics, Kastamonu Üniversitesi Tıp Fakültesi, Kastamonu, Turkey.

**Keywords:** Prostatic hyperplasia, Lower urinary tract symptoms, Nocturia, Blood pressure, Benign prostatic hyperplasia, Systolic pressure, Urgency

## Abstract

**BACKGROUND:**

Lower urinary tract symptoms significantly worsen quality of life. The hypothesis that they might lead to serious systolic blood pressure alterations through inducing sympathetic nervous activity has not been studied so far.

**OBJECTIVES:**

To investigate the relationship between benign prostate enlargement-related storage and voiding symptoms and systolic blood pressure.

**DESIGN AND SETTING:**

Cross-sectional single-center study on data from a hospital patient record system.

**METHODS:**

We evaluated the medical records of all consecutive patients with benign prostate enlargement-related lower urinary tract symptoms admitted between January 2012 and December 2017. Storage and voiding symptoms were assessed separately. International Prostate Symptom Score, uroflowmetry, postvoiding residual urine volume and systolic blood pressure were recorded. Pearson correlation and linear regression analysis were used.

**RESULTS:**

Positive correlations were found between systolic blood pressure and all of the storage symptoms. Among these, urgency had the most significant effect. There were 166 patients (41.4%) with urgency for urination, which increased mean systolic blood pressure from 124.88 mmHg (average value in elevated blood pressure group) to 132.28 mmHg (average value in stage-1 hypertension group). Hesitancy in urinating and feeling of incomplete bladder emptying had weak positive correlations with systolic blood pressure. There was a negative correlation between systolic blood pressure and intermittency of urination.

**CONCLUSIONS:**

With increasing numbers of urine storage symptoms, systolic blood pressure also increases, while the opposite occurs for voiding symptoms in patients with benign prostate enlargement. We conjecture that storage symptoms may lead to this increase through inducing sympathetic hyperactivity. Further prospective studies with larger groups are needed to confirm these findings.

## INTRODUCTION

Two types of lower urinary tract symptoms are associated with symptomatic benign prostate enlargement: storage symptoms and voiding symptoms. The detrimental effects of storage symptoms on patients’ quality of life, which have been recognized as a major burden on healthcare resources, are more significant than voiding symptoms.^1^ An association between lower urinary tract symptoms and cardiovascular hyperstimulus based on an overactive sympathetic nervous system has been reported.^2^ The filling and voiding cycles of the bladder trigger sympathetic activity and, thus, benign prostate enlargement-related storage and voiding symptoms stimulate autonomic hyperactivity.^3^^,^^4^^,^^5^

Since blood pressure elevation indicates sympathetic hyperactivity, this subject has become a focus of interest over recent years.^3^^,^^4^^,^^5^ The presence of hypertension as a component of metabolic syndrome has been recognized to play a role in the development of severe lower urinary tract symptoms.^6^ It has been shown that bladder dysfunction may occur in the presence of endothelial dysfunction in the pelvic vascular system. The mechanism is based on increased sympathetic activity, especially α1‐adrenoreceptor activity. This pathway is common for hypertension and severe lower urinary tract symptoms.^6^^,^^7^ Other studies have demonstrated that there is an association between benign prostate hyperplasia and hypertension via activation of insulin-like growth factor and increased sympathetic nervous system activity.^8^^,^^9^^,^^10^

So far, published studies have mainly focused on the International Prostate Symptom Score or, specifically, on the effects of nocturia on the blood pressure. ^4^^,^^5^ On the other hand, to the best of our knowledge, the relationship between each lower urinary tract symptom and systolic blood pressure (SBP) has not been studied. There is a serious lack of information about this topic.

## OBJECTIVES

Our aim was to investigate the association between SBP (especially stage-1 hypertension) and each benign prostate enlargement-related storage or voiding symptom, separately.

## METHODS

### Study design and ethics

Our study was designed as a cross-sectional evaluation of data extracted from our hospital’s patient record system. It was conducted after obtaining approval from the local ethics committee at our hospital (protocol number: 2018-05/58; date of approval: May 2, 2018), and in accordance with the ethical standards of the institutional and/or national research committee and with the 1964 Helsinki declaration and its later amendments or comparable ethical standards. Checklists compiled using the Strengthening the Reporting of Observational studies in Epidemiology (STROBE) recommendations were also used.

### Participants

The data of all 732 patients aged 50 years and over who presented benign prostate enlargement-related lower urinary tract symptoms and were admitted to our urology clinic between January 2012 and December 2017 were assessed.

Patients who presented stage-2 hypertension, diabetes, hyperlipidemia, uncontrolled hypothyroidism, obesity or metabolic syndrome; renal, cardiac, pulmonary, vascular, hepatic or psychiatric diseases; or sleep disorders or active urinary tract infections were excluded in order to eliminate other etiologies that might trigger sympathetic activity. Except for benign prostate enlargement, patients with histories of other urinary diseases, urethral manipulations and strictures or pelvic or cardiovascular surgery were excluded. Patients who were using antihypertensive drugs or alpha-blocker medications that might affect the autonomic nervous system were also excluded from the study in order to avoid the presence of misleading lower urinary tract symptoms and blood pressure measurements.

### Blood pressure assessment and hypertension classification

We used the 2017 guidelines of the American College of Cardiology/American Heart Association for blood pressure classification.^11^^,^^12^ Through these guidelines, all participants were firstly classified into four main groups based on their SBP and diastolic blood pressure (DBP) measurements: normotension (SBP < 120 mmHg and DBP ≤ 80 mmHg), elevated blood pressure (120 ≤ SBP < 130 mmHg and DBP > 80 mmHg), stage-1 hypertension (130 ≤ SBP < 140 mmHg or 80 ≤ DBP < 90 mmHg) and stage-2 hypertension (SBP ≥ 140 mmHg or DBP ≥ 90 mmHg.) Patients with stage-2 hypertension were excluded from the present study because these patients were using antihypertensive medication.

The patients thus selected were divided into three groups with regard to their SBP values. Patients with SBP of 110-119 mmHg (normotension), SBP of 120-129 mmHg (elevated blood pressure) and SBP of 130-139 mmHg (stage-1 hypertension). These were named the first, second and third group, respectively.

Basic vital signs and blood pressure measurements were part of our routine for every patient and these values were systematically recorded. During each visit, measurements were made after the patient had rested for at least five minutes in a warm room while sitting in a back-supported position.^12^ Systolic and diastolic blood pressure from the left brachial artery were measured using an automated blood pressure monitor (Tango, SunTech Medical, USA). Because blood pressure changes over time, it was measured at least twice on the same day with an interval of three minutes.^12^ After completion of the ultrasound, uroflowmetry and blood tests, the patients made a second visit approximately two weeks later. The average values for systolic blood pressure were recorded. No medication was given during this two-week period, so that the possible effects of alpha-blocker medications on systolic blood pressure were removed.

### Lower urinary tract symptom assessment and flow analysis

Following the International Continence Society’s classification scheme, patients who experienced urgency for urination, nocturia and high frequency of urination were classified as those with storage symptoms and patients who experienced intermittency of urination, hesitancy in urinating, feeling of incomplete bladder emptying and straining to urinate were classified as those with voiding symptoms. A validated Turkish-language version of the seven-item International Prostate Symptom Score was applied to assess subjective urinary symptoms. Each question was scored from 0 to 5. Total scores in the ranges of 0-7, 8-19 and 20-35 were classified as mild, moderate and severe, respectively.

Uroflowmetric analysis, including peak urinary flow rate, was recorded for every patient, followed by physical examination, digital rectal examination, urinalysis and prostate-specific antigen (PSA) measurement. Uroflowmetric measurements were performed using the Flowmaster Wireless Uroflowmeter (MMS-Medical Measurement Systems, Enschede, Netherlands). Prostate volume and postvoiding residual volume were measured using a 3.5-MHz transabdominal ultrasound probe (Acuson Sequoia 512; Siemens Medical Solution, Mountain View, CA, USA). This was positioned suprapubically in accordance with the ellipsoid formula, which consisted of multiplication together of the largest anteroposterior diameter (height, H), transverse diameter (width, W) and cephalocaudal diameter (length, L) with 0.524 (H × W × L × π/6).^13^ Among the patients with benign signs from rectal examination and a PSA value < 2.5 ng/ml, those with prostate sizes over 25 g or peak urinary flow rate values under 13 ml/s were classified as patients with benign prostate enlargement.

### Sample size calculation and statistical analysis

In order to achieve a power of 80.4% with 92.5% confidence interval for the statistical analysis, the sample size was designed to include at least 83 individuals in each of the three groups of SBP. The calculation yielded a total of 249 individuals.

Statistical analyses were performed using the IBM Statistical Package for the Social Sciences (SPSS), version 21 (IBM, Armonk, NY, USA). Continuous variables were presented as the mean ± standard deviation. The normality of continuous variables was evaluated using the Shapiro-Wilks test. Comparisons of continuous variables were performed using the independent-sample t test and one-way analysis of variance (ANOVA). Comparisons between pairs of categorical variables were made using chi-square analysis. The Pearson correlation coefficient was used to analyze associations between pairs of continuous variables. Multiple linear regression analysis was used to assess multivariate relationships between SBP and other variables. Candidate independent variables for multiple linear regression analysis were identified using univariate analysis. All tests were two-tailed and P-values of less than 0.05 were considered to be statistically significant.

## RESULTS

**Figure 1.**
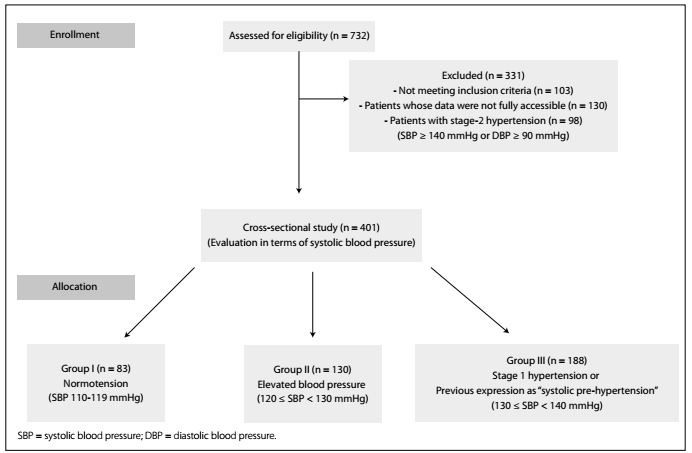
Flowchart of the study population.

It was determined that 401 patients were eligible for inclusion in this study. Figure 1 shows the flowchart for our study.

The mean age of all the patients was 57.26 ± 9.36 years. In the presence of urgency of urination, nocturia and high frequency of urination, the SBP values increased and the classification of the patients changed from “elevated blood pressure” to “stage-1 hypertension”. However, voiding symptoms did not lead to a similar effect on the systolic blood pressure (Table 1).

**Table 1.** Changes to systolic blood pressure according to lower urinary tract symptoms, post-voiding residual urine, prostate volume and age

Variable		Mean value of systolic blood pressure ± standard deviation	P-value
Urgency of urination	Absent (n = 235)	124.88 ± 7.50	< 0.001
	Present (n = 166)	132.28 ± 6.32	
Nocturia	Absent (n = 213)	125.06 ± 7.15	< 0.001
	Present (n = 188)	131.22 ± 7.48	
Urgency and nocturia	Absent (n = 296)	125.74 ± 7.26	< 0.001
	Present (n = 105)	134.16 ± 6.21	
High frequency of urination	Absent (n = 281)	126.84 ± 8.01	< 0.001
	Present (n = 120)	130.51 ± 7.08	
Intermittency of urination	Absent (n = 292)	129.02 ± 7.72	< 0.001
	Present (n = 109)	125.06 ± 7.75	
Hesitancy in urinating	Absent (n = 271)	129.04 ± 7.89	< 0.001
	Present (n = 130)	125.66 ± 7.50	
Feeling of incomplete bladder emptying	Absent (n = 306)	128.30 ± 7.96	0.104
	Present (n = 95)	126.79 ± 7.73	
Straining to urinate	Absent (n = 222)	130.10 ± 7.59	< 0.001
	Present (n = 179)	125.27 ± 7.51	
PVR (ml)	< 20 (n = 298)	128.24 ± 7.91	0.174
	21-50 (n = 55)	126.09 ± 8.12	
	> 50 (n = 48)	128.23 ± 7.64	
Prostate volume (g)	25-40 (n = 188)	128.38 ± 8.08	0.305
	> 40 (n = 213)	127.56 ± 7.77	
Age	R = -0.068		0.174

PVR = post-voiding residual urine.

Continuous variables are given as mean ± standard deviation (independent-sample t test). Correlation between age and systolic blood pressure was analyzed using Pearson correlation analysis.

When the cutoff value was set at 40 g, the increase in prostate volume did not lead to a significant change in SBP (P = 0.305). Similarly, no statistically significant relationship between postvoiding residual volume and SBP was found (P = 0.174) (Table 1). Among the seven symptoms, urgency for urination was found to have the most significant effect on SBP with an increase of 6.55 mmHg (P < 0.001). That effect was followed by nocturia, high frequency of urination, hesitancy in urinating and feeling of incomplete bladder emptying with increases of 4.63, 3.33, 2.37 and 1.67 mmHg, respectively. While straining to urinate had no effect on SBP, intermittency of urination led to a decrease in SBP of 1.78 mmHg (Table 2). The distributions of each group were presented in relation to the presence of lower urinary tract symptoms and the severity of the International Prostate Symptom Score (Table 3). This analysis did not identify any significant effects on SBP from the severity of the International Prostate Symptom Score and the peak urinary flow rate (Tables 2 and 3). As the number of storage symptoms increased, the SBP value also increased (P < 0.001, r = 0.536). On the contrary, as the number of voiding symptoms increased, the SBP value decreased (P < 0.001, r = -0.327).

**Table 2.** Effects of urinary symptoms on systolic blood pressure (linear regression analysis)

Covariates	t-value	β (95% confidence interval)	P-value
Constant	71.38	124.42 (120.99 to 127.85)	< 0.001
Urgency of urination	8.49	6.55 (5.03 to 8.06)	< 0.001
Nocturia	6.03	4.63 (3.12 to 6.14)	< 0.001
High frequency of urination	4.46	3.33 (1.86 to 4.80)	< 0.001
Intermittency of urination	-2.47	-1.78 (-3.75 to -0.43)	0.014
Hesitancy in urinating	2.57	2.37 (0.55 to 4.18)	0.011
Feeling of incomplete bladder emptying	1.80	1.67 (-0.15 to 3.49)	0.016
Straining to urinate	-1.67	-1.71 (-3.73 to 0.31)	0.096
Qmax	-1.91	-0.14 (-0.27 to 0.01)	0.057
IPSS-moderate	-0.54	-0.44 (-2.05 to 1.16)	0.588
IPSS-severe	0.75	-0.76 (-1.23 to 2.75)	0.454

Qmax = peak urinary flow rate; IPSS = International Prostate Symptom Score.

“IPSS-mild” was taken as the reference category, and so the categories of “IPSS-moderate” and “IPSS-severe” were evaluated in relation to the reference value.

**Table 3.** Distribution of groups in terms of presence of urinary symptoms and IPSS severity

Variable		Systolic blood pressure			P-value
		110-119 mmHg (n = 83)	120-129 mmHg (n = 130)	130-139 mmHg (n = 188)	
Urgency of urination	Absent, n (%)	71 (85.8)	99 (76.2)	65 (34.6)	< 0.001^*^
	Present, n (%)	12 (14.5)	31 (23.8)	123 (65.4)	
Nocturia	Absent, n (%)	59 (71.1)	90 (69.2)	64 (34.0)	< 0.001^*^
	Present, n (%)	24 (28.9)	40 (30.8)	124 (66.0)	
High frequency of urination	Absent, n (%)	75 (90.4)	91 (70.0)	115 (61.2)	< 0.001^*^
	Present, n (%)	8 (9.6)	39 (30.0)	73 (38.8)	
Intermittency of urination	Absent, n (%)	49 (59.0)	90 (69.2)	153 (81.4)	< 0.001^*^
	Present, n (%)	34 (41.0)	40 (30.8)	35 (18.6)	
Hesitancy in urinating	Absent, n (%)	47 (56.6)	77 (59.2)	147 (78.2)	< 0.001^*^
	Present, n (%)	36 (43.4)	53 (40.8)	41 (21.8)	
Feeling of incomplete bladder emptying	Absent, n (%)	62 (74.7)	89 (68.5)	155 (82.4)	0.015^*^
	Present, n (%)	21 (25.3)	41 (31.5)	33 (17.6)	
Straining to urinate	Absent, n (%)	32 (38.6)	56 (43.1)	134 (71.3)	< 0.001^*^
	Present, n (%)	51 (61.4)	74 (56.9)	54 (28.7)	
IPSS	Mild, n (%)	28 (33.7)	34 (26.2)	78 (41.5)	0.065
	Moderate, n (%)	38 (45.8)	67 (51.5)	71 (37.8)	
	Severe, n (%)	17 (20.5)	29 (22.3)	39 (20.7)	

IPSS = International Prostate Symptom Score.

*Chi-square test.

In a subgroup analysis that was composed of 33 patients who only had storage symptoms and 27 patients who only had voiding symptoms, SBP was found to be significantly higher in the patients who only had storage symptoms (135.21 ± 6.10 versus 128.04 ± 7.62 mmHg) (P < 0.001).

## DISCUSSION

Many studies have investigated factors such as age and health-related, physical, psychiatric, lifestyle, socioeconomic and metabolic factors that might show associations with storage and voiding symptoms.^3^^,^^14^^,^^15^^,^^16^^,^^17^^,^^18^ Some studies have found divergent results regarding the factors associated with storage and voiding symptoms.^4^^,^^5^ Previous epidemiological studies have investigated links between sympathetic overactivity and lower urinary tract symptoms.^4^^,^^5^^,^^19^^,^^20^^,^^21^^,^^22^ Recently, the effects of lower urinary tract symptoms on sympathetic nervous system activity have been drawing attention.

Some studies have demonstrated that pathophysiological similarities and common pathways exist between lower urinary tract symptoms and autonomic nervous system hyperactivity.^20^^,^^21^ Animal models have demonstrated that autonomic nervous system activity is an important determinant of prostate growth and, thus, that it is associated with lower urinary tract symptoms.^20^^,^^21^ McVary et al. reported that changes to the American Urological Association Symptom score, Benign Prostatic Hyperplasia Impact Index score and Quality of Life score had significant effects on systolic and diastolic blood pressure.^21^ They found a positive correlation between total symptom scores and blood pressure, although they did not evaluate the effect of each symptom separately. Similarly, in another study, International Prostate Symptom Score and peak urinary flow rate values were identified as positively related variables for diastolic blood pressure, and this finding was associated with increased sympathetic activity.^22^

Systolic or diastolic blood pressure has been presented as a determinant of nocturia.^23^ The circadian rhythm of blood pressure has been investigated among patients with benign prostate enlargement, and presence of nocturia has been found to be an independent risk factor for non-dipper hypertension. Its presence has also been reported to be a poor prognostic factor for cardiovascular morbidity and mortality.^24^

Martin et al. evaluated many factors associated with uncomplicated storage and voiding symptoms. None of the storage and voiding symptoms were found to be significant in terms of SBP and DBP.^3^ Based on the effects of thyroid dysfunction on autonomic nervous system activity, they noted that increased modulation of beta-2 adrenergic receptors may lead to voiding symptoms.^25^ On the other hand, if their conjecture were correct, this reasoning should also explain a similar relationship between SBP and both voiding and storage symptoms.

According to a rodent model, chronic intermittent hypoxia causes increased HIF-1 alpha synthesis. This alerts the sympathetic neurons and stimulates the release of epinephrine, norepinephrine and other catecholamines. As a result, blood pressure increases.^26^ This mechanism is very similar to benign prostate enlargement. Prostate hyperplasia gives rise to inadequate blood flow to prostate cells. It activates chronic hypoxia and ischemia, and many mediators such as HIF-1 alpha, VEGF and TGF beta start to be released. The association between sympathetic systemic activity and lower urinary tract symptoms can be explained by this mechanism.^27^ In another animal model, it was shown that hypertensive rats exhibited symptoms of voiding dysfunction and had increased presence of sympathetic neurotransmitters. Alpha-1 sympathetic hyperactivity is a well-known dynamic component of benign prostate enlargement because it increases the smooth muscle tone of the prostate and causes bladder outlet obstruction.^22^

According to Jang et al., in patients with sympathetic hyperactivity, alpha blockers were less effective.^28^ They commented that sympathetic hyperactivity was a negative prognostic factor for progression of benign prostate enlargement. However, this correlation was only observed in relation to the total International Prostate Symptom Score. They measured the patients’ heart rate variability to determine sympathetic activity, although all of the patients were normotensive.

Our study differs from the previous studies because we investigated each voiding and storage symptom separately, in terms of their association with SBP. We found that storage symptoms were more significant determinants than voiding symptoms, in terms of stage-1 systolic hypertension. Even if these patients have blood pressure values that do not require any medication, they still could experience cardiac problems in their future lives.

According to the 2007 guidelines of the European Society of Cardiology/European Society of Hypertension, and the Seventh Report of the Joint National Committee on Prevention, Detection, Evaluation and Treatment of High Blood Pressure, prehypertension was classified as 130 ≤ SBP < 140 mmHg and/or 80 ≤ DBP < 90 mmHg.^12^^,^^29^ It was recommended that cardiologists should start administering antihypertensive medication when prehypertension is detected. Since then, systolic prehypertension has been the focus of discussion and research. In the 2014 guidelines, the recommendation was that cardiologists should not start treatment with medication based on the presence of systolic prehypertension; instead, very close follow-up was recommended, given that these patients were at very high risk of harboring or developing serious cardiovascular diseases.^12^

The 2017 guidelines of the American College of Cardiology/American Heart Association changed the definition of prehypertension, to create two different stages: elevated blood pressure (120 ≤ SBP < 130 mmHg and DBP > 80 mmHg) and stage-1 hypertension (130 ≤ SBP < 140 mmHg or 80 ≤ DBP < 90 mmHg).^11^ Thus, the term “systolic prehypertension” was replaced by “stage-1 systolic hypertension”. The alert value requiring follow-up became 130 ≤ SBP < 140 mmHg.

Patients with benign prostate enlargement experiencing lower urinary tract symptoms, who did not need antihypertensive medication, were included in our study. We divided them into subgroups in terms of SBP. Although we did not detect any statistically significant correlation between the International Prostate Symptom score, peak urinary flow rate and SBP when lower urinary tract symptoms were examined individually, the other six symptoms except for straining to urinate were found to have significant effects on SBP. Among these six symptoms, urgency of urination, nocturia and high frequency of urination had the strongest effects on SBP and shifted the blood pressure stage from “elevated blood pressure” to “stage-1 hypertension”. The contributions of these symptoms to increased SBP were 6.55, 4.63 and 3.33 mmHg, respectively and these increases were statistically significant. Considering that, according to the new American College of Cardiology/American Heart Association classification, the differences between the groups are only 10 mmHg, and that such differences in SBP (especially in the case of increases) change the therapeutic approach that physicians take, we believe that these results are clinically significant as well.

Hypertension and benign prostatic hyperplasia (BPH) are common age-related diseases. These two pathophysiological conditions coincide in about 25-30% of men over 60 years old.^30^ Hypertension increases the risk of moderate-to-severe lower urinary symptoms by 1.5-fold.^31^ Conversely, we observed that severe lower urinary symptoms induced higher systolic blood pressure. Because all the patients in our study had blood pressure values under 140 mmHg, there was no need for medication.

We demonstrated that as the number of storage symptoms increased, the SBP value also increased. As the number of voiding symptoms increased, the SBP value decreased. Sympathetic activity was not directly measured in this study, but our findings were compatible with those of other studies that measured sympathetic activity and suggested that sympathetic activity could be triggered via storage symptoms.^4^^,^^20^^,^^21^^,^^28^

When the cutoff value was set at 40 g, we observed that the increase in prostate volume did not lead to a significant change in SBP. This was expected, because there is no direct correlation between prostate volume and the severity of lower urinary tract symptoms. Prostatic hyperplasia is a histological phenomenon and does not always have to cause obstruction, whereas lower urinary tract symptoms are a mixture of neuromuscular changes that are assumed to interact with the autonomic nervous system. Prostate enlargement of solely non-obstructive nature has no direct connection with the neuronal changes that play a major role in lower urinary tract symptoms. It is assumed that prostate enlargement alone does not have an impact on the autonomic nervous system unless it is symptomatic.

We did not find any relationship between postvoiding residual volume and SBP. This may be explained by the low residual urine volumes. Most of the patients were within the normal range and hardly any were above the generally well-accepted limit of 50 ml. At values under 50 ml, there are no SBP-related changes, possibly because there is no irritation effect on the bladder. At values above 50 ml, compensation due to continuous sympathetic stimulation develops and there is inhibition of nerve conduction.^19^^,^^32^

Because heart rate variability can be assessed as a non-invasive indicator of sympathetic nervous system function, some researchers have used it to analyze sympathetic activity.^4^^,^^28^ Thus, they synchronously evaluated the relationship between heart rate variability and lower urinary tract symptoms because heart rate variability reflected spontaneous changes in autonomic activity. In another study, changes to SBP and DBP were recorded one and five minutes after a tilt table test, respectively, to assess alterations in autonomic activity. Thus, the response to circulatory stress was evaluated via the tilt table.^21^

One limitation of our study was that we did not measure the variability of sympathetic activity via heart rate variability or a stress tilt test. We defend our hypothesis based on the findings in the literature. If we had been able to prove that variability in sympathetic activity was present**,** the power of our study would have become higher, but this was not possible because of our study design. Another limitation of our study was the small number of patients.

To our knowledge, this was the first study to evaluate voiding and storage symptoms separately in terms of their associations with SBP, in patients with benign prostate enlargement-related lower urinary tract symptoms.

## CONCLUSIONS

We presume that storage symptoms are directly related to systolic blood pressure levels. When each symptom was analyzed individually, urgency of urination, nocturia and high frequency of urination were found to be correlated with the most significant rises in systolic blood pressure. We also found that the combination of urgency of urination and nocturia had an adjuvant effect and together increased the systolic blood pressure more than each symptom did alone. In accordance with previous studies, we hypothesize that storage symptoms provoke sympathetic system activity and may manifest as stage-1 hypertension, which is an important precursor for cardiovascular diseases. Further prospective studies with larger patient groups are needed to prove this relationship and to show whether treatment of storage symptoms may help in treating stage-1 systolic hypertension.
